# Synthesis of a well-dispersed CaFe_2_O_4_/g-C_3_N_4_/CNT composite towards the degradation of toxic water pollutants under visible light†

**DOI:** 10.1039/c9ra05005a

**Published:** 2019-08-15

**Authors:** Fei Liu, Shaocan Dong, Zhaoxiang Zhang, Xiaqing Li, Xiaodong Dai, Yanping Xin, Xuewu Wang, Kun Liu, Zhenhe Yuan, Zheng Zheng

**Affiliations:** Shengli College, China University of Petroleum Dongying Shandong 257061 China lf88888888lz@sina.com; Shengli Oilfield Company Postdoctoral Research Station, Sinopec Dongying Shandong 257000 China; Petroleum Engineering Technology Research Institute of Shengli Oilfield Company, SINOPEC Dongying Shandong 257000 China

## Abstract

Herein, we fabricated a ternary photocatalyst composed of CaFe_2_O_4_, multiwalled carbon nanotubes (CNTs) and graphitic carbon nitride (g-C_3_N_4_) *via* a simple hydrothermal route. CaFe_2_O_4_ acted as a photosensitizer medium and the CNT acted as a co-catalyst, which remarkably enhanced the photocatalytic performances of g-C_3_N_4_ towards the degradation of hexavalent chromium (Cr(vi)) and the antibiotic tetracycline (TC) under visible light irradiation. To investigate the morphological and topological features of the photocatalyst, field-emission scanning electron microscopy (FE-SEM) and transmission electron microscopy (TEM) analyses were performed. The surface properties and oxidation state of the CaFe_2_O_4_/g-C_3_N_4_/CNT composite were determined by X-ray photoelectron spectroscopy (XPS). The recombination rate of the charge carriers and the band gap values of the as-synthesized catalysts were analyzed by photoluminescence spectroscopy (PL) and diffused reflectance spectroscopy (UV/Vis DRS) studies, respectively. Besides the degradation reactions, the high hydrogen production rate of 1050 μmol h^−1^ under visible light using the CaFe_2_O_4_/g-C_3_N_4_/CNT composite loaded with 5 wt% CNT was observed. The superior photocatalytic performances of the CaFe_2_O_4_/g-C_3_N_4_/CNT composite can be ascribed to the effective heterojunction formed between g-C_3_N_4_ and the CaFe_2_O_4_ matrix, in which the CNT act as a conducting bridge in the system, promoting the production of photoinduced charge carriers in the semiconductor system. Finally, the plausible photocatalytic mechanism towards the degradation of pollutants and hydrogen production was discussed carefully.

## Abstract 2

Herein, we fabricated a ternary photocatalyst composed of CaFe_2_O_4_, multiwalled carbon nanotubes (CNTs) and graphitic carbon nitride (g-C_3_N_4_) *via* a simple hydrothermal route.

## Graphical abstract

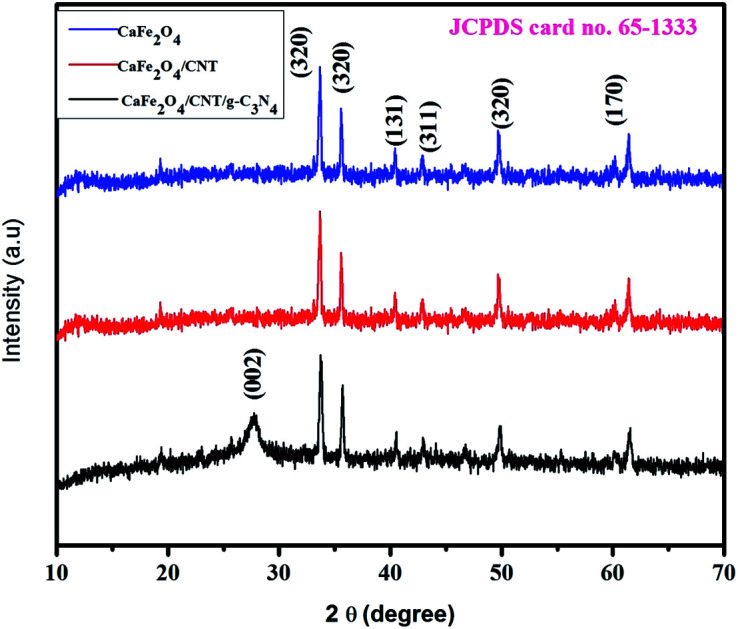

## 1. Introduction

In the past few decades, the reckless release of organic pollutants in water bodies has increased greatly, which cause severe environmental problems and health risks.^1,2^ Accordingly, the photocatalysis process using semiconductor photocatalysts has been proposed as a green technology to degrade the organic pollutants in water bodies, even at trace levels.^3^ Thus, semiconductor materials, such as TiO_2_, SnO_2_, and ZnO, have been investigated by various researchers owing to their low cost and ease of availability.^4^ However, their foremost drawbacks are their wide band gap energy, high recombination, and low visible light response, which limit their large-scale production in industry.

Recently, a polymeric semiconductor, g-C_3_N_4_, has emerged with visible light response, excellent quantum efficiency and photochemical stability, presenting a competitive candidate for the degradation of dyes, NO_*x*_ removal, CO_2_ reduction, and water-splitting reactions.^5^ Unfortunately, the pure g-C_3_N_4_ semiconductor exhibits a sluggish performance due to its high recombination rate and poor absorption ability in the entire visible light region. Furthermore, its low quantum efficiency and turnover number (TON) are also considered a major obstacle.^6^ Thus, tremendous efforts have been made to resolve these problems, and a system mainly composed of well-matched semiconductors will be a good way to overcome these issues.^7^ The heterojunction between highly visible light active semiconductors with suitable band gap values is favorable for enhanced charge separation efficiency in g-C_3_N_4_ and dramatically enhances its light absorption ability in the entire visible region.^8^

Thus, calcium ferrite (CaFe_2_O_4_), as p-type visible light-active photocatalyst, has been investigated due to its low cost and toxicity with narrow band gap energy towards the degradation of pollutants in both the gaseous and liquid phase.^9^ Nevertheless, its major drawbacks such as inefficient hole transfer property, high recombination rate, and charge mobility result in a sluggish performance.^10^ Therefore, the CaFe_2_O_4_ photocatalysts suffered severely during water-splitting reactions and the degradation of pollutants. To solve these issues, Luo *et al.* developed a novel CaFe_2_O_4_/Bi_2_O_3_ composite *via* a simple wet chemical route. The photocatalytic properties of the CaFe_2_O_4_/Bi_2_O_3_ composite was evaluated through the degradation of malachite green (MG) dye under visible light irradiation.^11^ The higher photocatalytic efficiency can be attributed due to the improved separation of photogenerated electron–hole pairs through the incorporation of Bi_2_O_3_, which can be beneficial for a higher degradation performance. Liu *et al.* prepared a carbon-modified CaFe_2_O_4_ composite towards the degradation of methylene blue dye.^12^ The presence of carbon in CaFe_2_O_4_ greatly influenced the degradation rate of CaFe_2_O_4_ due to its excellent adsorption behavior.

Therefore, it is essential to develop simple and cost-effective methods to improve the photocatalytic ability of both CaFe_2_O_4_ and g-C_3_N_4_ without sacrificing their crystalline nature and structural stability. CNT are outstanding 1D nano-sized materials with abundant surface hydroxyl and carboxyl groups, which enable their solubility in polar solvents.^13^ Thus, anchoring CNT on the surface of CaFe_2_O_4_ and g-C_3_N_4_ can lead to a superior performance than that of the CaFe_2_O_4_ and g-C_3_N_4_ binary system. Besides, CNT exhibit a high specific surface area, excellent electron transfer ability, and can act as a noble metal-free co-catalyst in heterojunction systems.^14^ The CNT-enabled heterojunction system can afford extra active sites for the adsorption of target pollutants in degradation reactions. Inspired by this mechanism and to further improve the photo-degradation efficiency of both CaFe_2_O_4_ and g-C_3_N_4,_ herein, we demonstrate a simple method for the development of a ternary photocatalyst.^15^ Upon the incorporation of CNT, the photocatalytic efficiency was significantly improved towards the hydrogen evolution reaction, and Cr(vi) and TC degradation to a great extent.

## 2. Experimental

### 2.1. Materials

All the chemicals used in this work were of analytical reagent (AR) grade. Dicyandiamide, melamine (C_3_H_6_N_6_), calcium nitrate tetrahydrate (Ca(NO_3_)_2_·4H_2_O), iron(iii) nitrate nonahydrate (Fe(NO_3_)_3_·9H_2_O), potassium dichromate, tetracycline hydrochloride, glycerol, ethylene glycol, citric acid, ethanol, methanol and MWCNT were obtained from Sigma-Aldrich Corporation. Deionized (DI) was used throughout the synthesis and photocatalytic process.

### 2.2. Preparation of g-C_3_N_4_

g-C_3_N_4_ was prepared *via* a simple calcination route. In a typical synthesis, a stoichiometric amount of dicyandiamide and melamine (1 : 1 ratio) was added to a silica crucible and calcined at 550 °C for 4 h under a nitrogen atmosphere. Finally, the silica crucible was cooled naturally to obtain porous g-C_3_N_4_ sheets.

### 2.3. Preparation of CaFe_2_O_4_

CaFe_2_O_4_ was synthesized as follows method: a calculated amount of Ca(NO_3_)_2_·4H_2_O, Fe(NO_3_)_3_·9H_2_O, glycerol, ethylene glycol, and citric acid was mixed under ultrasound irradiation and stirred for 1 h at 80 °C. The obtained red color fluffy product was further calcined at 850 °C for 3 h under an air atmosphere to obtain the pure-phase CaFe_2_O_4_.

### 2.4. CaFe_2_O_4_/g-C_3_N_4_/CNTs composite

A simple hydrothermal route was adopted to synthesize the CaFe_2_O_4_/g-C_3_N_4_/CNT composite. Typically, the CNT and as-synthesized g-C_3_N_4_ were dispersed in 20 mL of DI water with the help of ultrasonication for 2 h. Then, a calculated weight percentage of CaFe_2_O_4_ powder was mixed with the above solution and transferred to a Teflon-lined autoclave, which was heated at 120 °C for 12 h. The final brown-colored precipitate was washed with DI water repeatedly and vacuum dried at 70 °C overnight.

### 2.5. Characterization

The CaFe_2_O_4_/g-C_3_N_4_/CNT composite was analyzed *via* X-ray diffraction (XRD) on a PANalytical X-ray diffractometer using CuKα radiation (*λ* = 1.5418 Å) in the 2*θ* range of 10–70°. The elemental purity and surface properties of the CaFe_2_O_4_/g-C_3_N_4_/CNT composite was examined *via* X-ray photoelectron spectroscopy (XPS) on an ESCALAB X-ray photoelectron spectrometer using MgKα radiation. The morphology and microstructures of the prepared samples were recorded *via* TEM (Tecnai G2 F20 transmission electron microscope), and field-emission scanning electron microscopy using an EM, Quanta FEG 250. UV-Vis diffuse reflectance spectra (DRS) of the samples were analyzed an a UV/Vis spectrophotometer (Jasco-V750 Japan) in the wavelength range of 200–900 nm using BaSO_4_ as an internal standard. Photoluminescence (PL) spectra were measured using a fluorescence spectrophotometer (F-7000, Japan).

### 2.6. Photocatalytic degradation reactions

The aqueous phase degradation of Cr(vi) and TC was performed in a quartz reactor under visible light irradiation (*λ* ≥ 420 nm) generated by a 300 W Xe lamp. The distance between the test solution and the lamp was fixed at 15 cm. In the degradation process, 0.1 g of powdered photocatalyst was mixed in 100 mL of Cr(vi) and TC with a concentration of 10 ppm. Before visible light irradiation, the suspension was kept in the dark with constant stirring for 20 min to attain equilibrium between the pollutants and photocatalyst. Subsequently, the degraded suspension was collected at given time periods and the absorbance maxima were analyzed by a UV-vis spectrophotometer.

### 2.7. Photocatalytic H_2_ evolution reactions

To measure the hydrogen production rate, the following procedure was adopted. Exactly 50 mg of CaFe_2_O_4_/g-C_3_N_4_/CNT composite was dispersed in 75 mL of 15 vol% lactic acid solution, and then purged with N_2_ for 30 min to remove the oxygen in the reactor vessel. A 4 W LED was applied as the visible-light source and continuous stirring was maintained to keep the photocatalyst suspended in the medium. The amount of hydrogen produced was measured by a gas chromatograph with a TCD detector. The AQY of the CaFe_2_O_4_/g-C_3_N_4_/CNTs was calculated using the following equation:
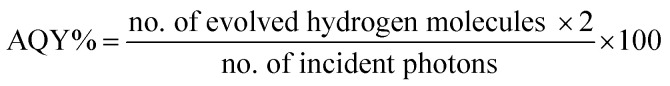


## 3. Results and discussion

**Fig. 1:**
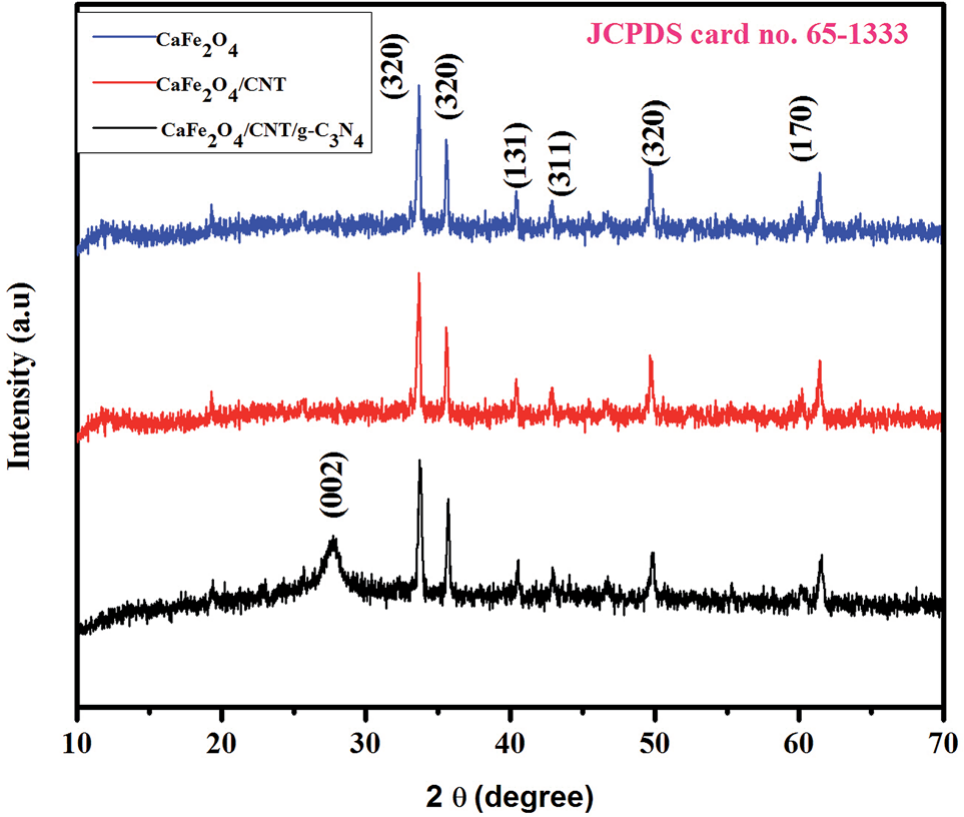
XRD patterns of the as-prepared CaFe_2_O_4_ and CaFe_2_O_4_/g-C_3_N_4_/CNT composite.

**Fig. 2:**
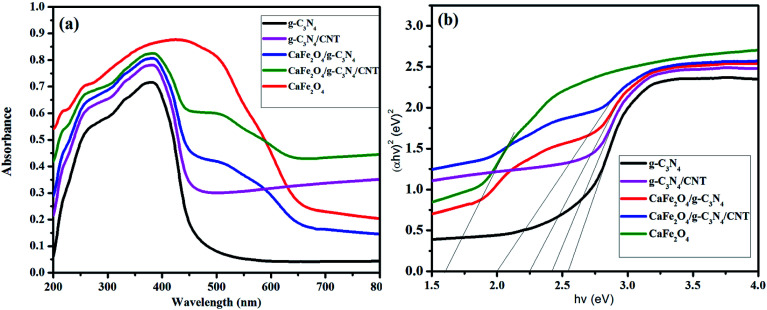
(a) UV-vis diffuse reflectance spectra of CaFe_2_O_4_, g-C_3_N_4_, g-C_3_N_4_/CNTs, and CaFe_2_O_4_/g-C_3_N_4_/CNT composite. (b) Optical band gap energy, *E*_g_, of CaFe_2_O_4_, g-C_3_N_4_, g-C_3_N_4_/CNTs, and CaFe_2_O_4_/g-C_3_N_4_/CNT composite.

Fig. 1 shows the XRD patterns of g-C_3_N_4,_ CaFe_2_O_4_, and CaFe_2_O_4_/g-C_3_N_4_/CNT composite. Two apparent distinguishing peaks of g-C_3_N_4_ appeared at the 2*θ* values of 13.1° and 27.8°, which can be perfectly matched with the JCPDS card number 87-1526.^16^ All the peaks of CaFe_2_O_4_ correspond to the orthorhombic crystal phase and matched the standard card (JCPDS 32-0168).^17^ The strong interference peak of g-C_3_N_4_ at 27.5 was observed in the CaFe_2_O_4_/g-C_3_N_4_/CNT composite, confirming the successful formation of the heterojunction. Furthermore, the characteristic peaks of the CNT were not observed in the system, which can be attributed to their low concentration and poor crystallinity. Thus, the optical properties of the as-synthesized catalyst samples were recorded by UV-vis DRS analysis. Fig. 2a presents the typical UV-vis DRS spectrum of g-C_3_N_4,_ CaFe_2_O_4_ and CaFe_2_O_4_/g-C_3_N_4_/CNT composite. It can be observed that the absorption maximum of CaFe_2_O_4_ is about 466 nm, while the pure g-C_3_N_4_ exhibits poor visible light absorption about 470 nm. From the Tauc plot, the band gap energies of CaFe_2_O_4_ and g-C_3_N_4_ were calculated to be 1.55 eV and 2.57 eV, respectively. Comparing the absorption maxima of g-C_3_N_4_, g-C_3_N_4_/CNT and g-C_3_N_4_/CaFe_2_O_4_ nanocomposites, the CaFe_2_O_4_/g-C_3_N_4_/CNT composite exhibited a significant red shift with the characteristic absorption edge at 585 nm and band gap energy of 2.02 eV, as shown in Fig. 2b. This may be attributed to the successful formation of an active heterojunction between the g-C_3_N_4_ and CaFe_2_O_4_ interface and strong electron conducting nature of CNT.^18^ Moreover, the enhanced visible light absorption confirmed that the CaFe_2_O_4_/g-C_3_N_4_/CNT composite can absorb the visible light effectively, which is favorable for the production of hydrogen and degradation of pollutants.^19^

**Fig. 3:**
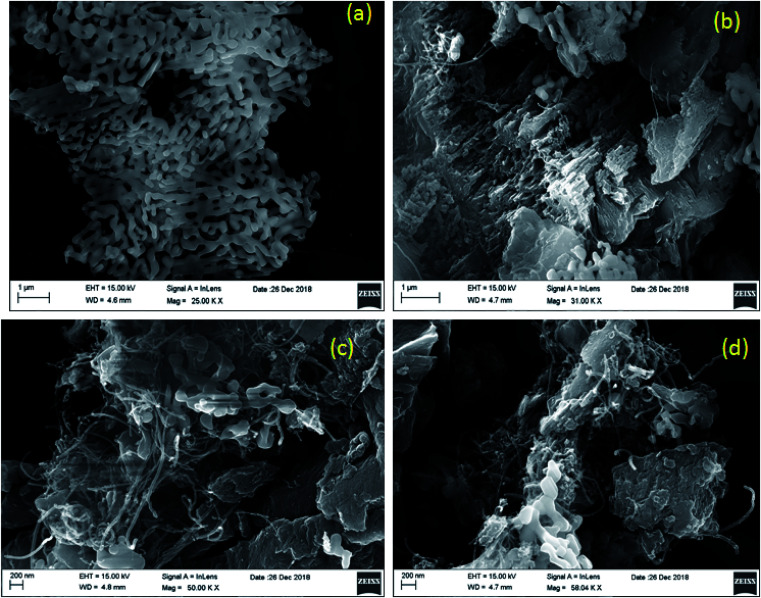
(a–d) FE-SEM images of CaFe_2_O_4_ and CaFe_2_O_4_/g-C_3_N_4_/CNT composite.

**Fig. 4:**
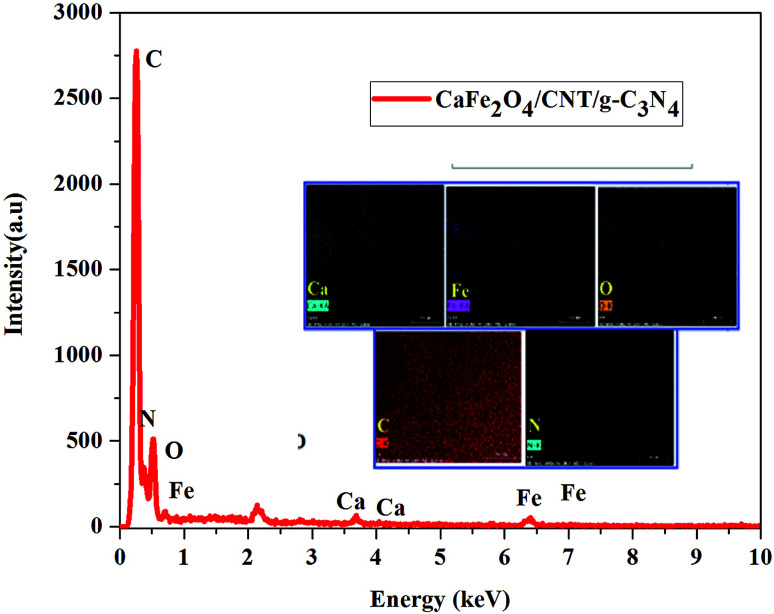
EDS and elemental mapping analysis of the CaFe_2_O_4_/g-C_3_N_4_/CNT composite.

The morphologies of the as-synthesized composites were analyzed by FE-SEM and TEM analysis. The SEM images in Fig. 3a–d show the morphology and structure of CaFe_2_O_4_ and the CaFe_2_O_4_/g-C_3_N_4_/CNT composite. Fig. 3a shows that the CaFe_2_O_4_ particles are well-dispersed with an average size of 200–300 nm.^20^Fig. 3b–d show the FE-SEM images of the CaFe_2_O_4_/g-C_3_N_4_/CNT composite, which contains CaFe_2_O_4_, CNT and g-C_3_N_4_ nanosheets, respectively. Furthermore, the FE-SEM images reveal that g-C_3_N_4_ exhibited a porous structure with few aggregations. The tube-like CNT were interlinked effectively on the surface of the g-C_3_N_4_ and CaFe_2_O_4_ matrices.^21^ The existence of this heterojunction was further confirmed by the representative elemental mapping images (EDS) in Fig. 4, which show an even distribution of carbon (C), nitrogen (N), calcium (a), oxygen (O) and iron (Fe).

**Fig. 5:**
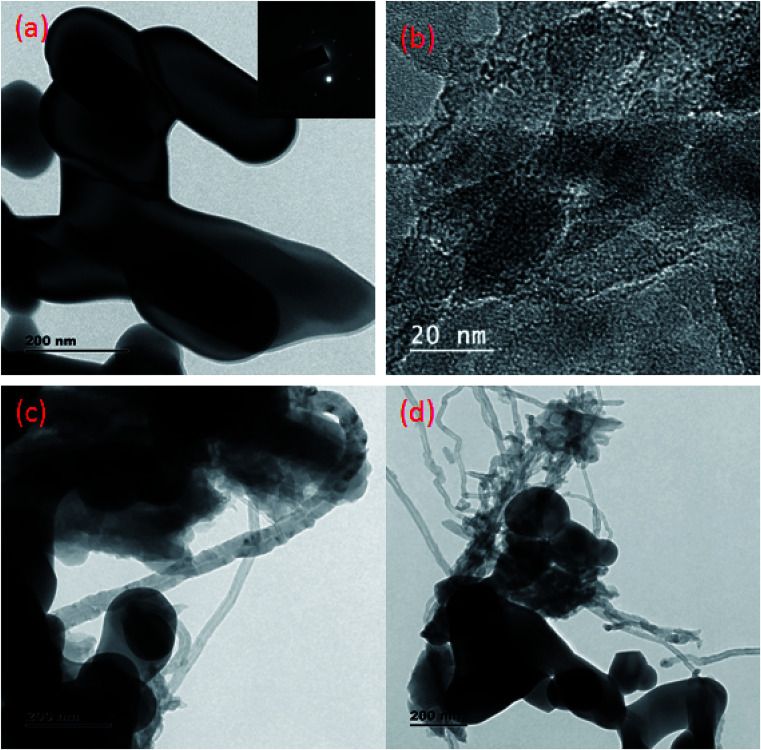
(a) TEM images of CaFe_2_O_4_ (b) g-C_3_N_4_ and (c and d) CaFe_2_O_4_/g-C_3_N_4_/CNT composite.

**Fig. 6:**
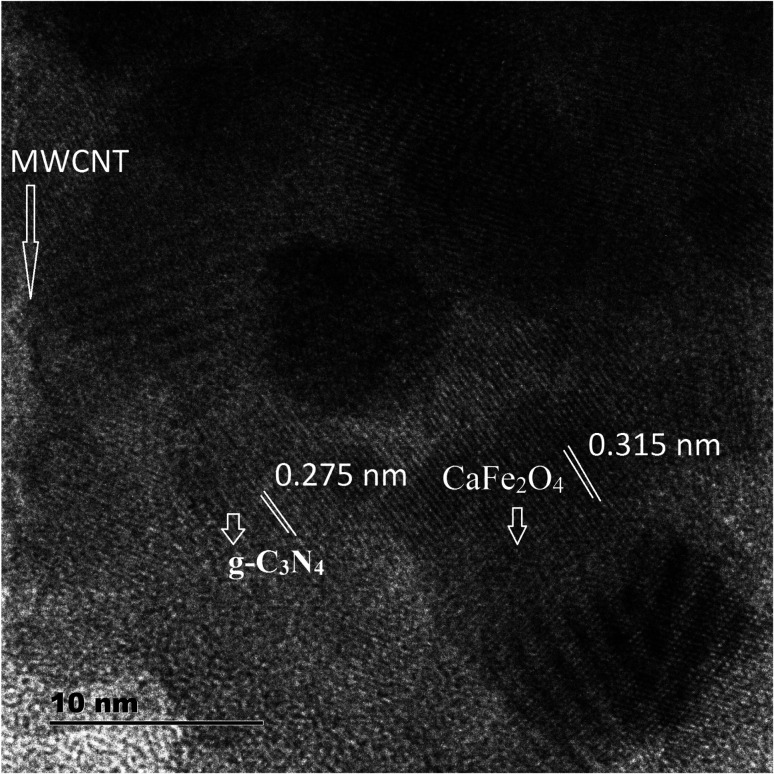
HRTEM images of the CaFe_2_O_4_/g-C_3_N_4_/CNT composite.

Further, to investigate the microstructure of the obtained samples, TEM characterization was performed. Fig. 5a–d present the typical TEM images of CaFe_2_O_4_, g-C_3_N_4_, and the CaFe_2_O_4_/g-C_3_N_4_/CNT composite. For the composite, grey part can be assigned to the sheet-like g-C_3_N_4_, and the dark particles can be assigned to CaFe_2_O_4_ nanoparticles, which were dispersed effectively on the exterior surface of g-C_3_N_4_.^22^ Furthermore, the TEM images display the existence of a heterojunction stuck between g-C_3_N_4_ and CaFe_2_O_4_, and the tubular CNT acting as a connecting bridge between these materials. In the SAED patterns, the lattice fringes have a spacing of 0.231 nm, which is consistent with the (131) planes of CaFe_2_O_4_ (JCPDS 32-0168). Thus, these results are in accordance with the XRD analysis. Upon further analysis of the HRTEM images, as shown in Fig. 6, close contact interfaces between CaFe_2_O_4_, g-C_3_N_4_ and CNT were observed. From the TEM results, it can be concluded that an apparent interface was formed between the g-C_3_N_4_, CNT, and CaFe_2_O_4_ matrices. Therefore this ternary CaFe_2_O_4_/g-C_3_N_4_/CNT composite is promising for the transport of charge carriers towards the degradation of Cr(vi) and TC.^23^

**Fig. 7:**
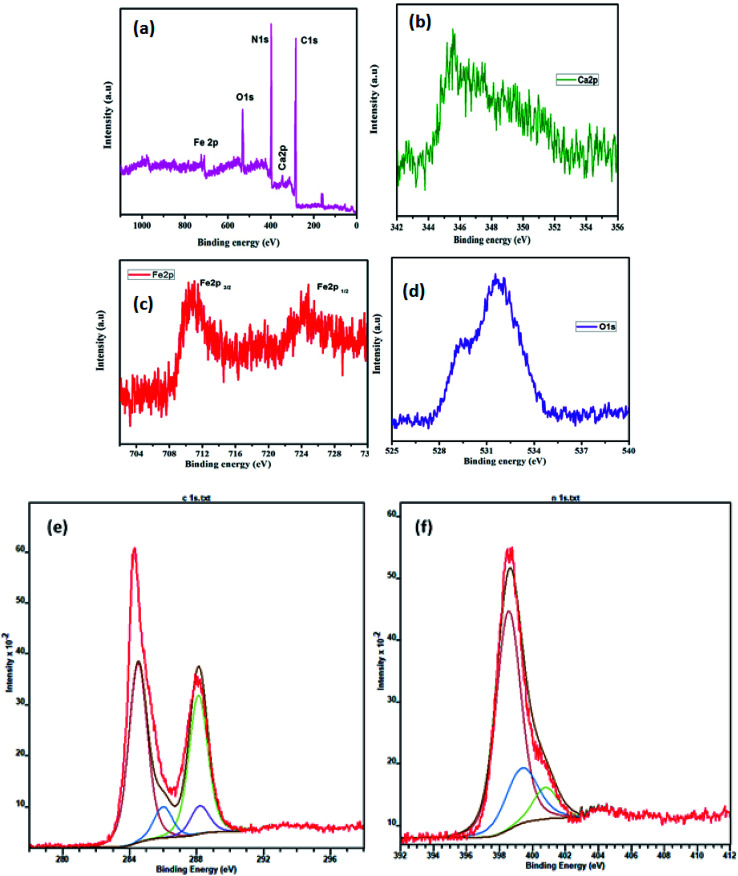
XPS spectra of the as-prepared CaFe_2_O_4_/g-C_3_N_4_/CNT composite: (a) survey scan, (b) Ca 2p, (c) Fe 2p, (d) O 1s, (e) C 1s, and (f) N 1s.

The chemical binding energies and oxidation states of the CaFe_2_O_4_/g-C_3_N_4_/CNT composite were confirmed by XPS (Fig. 7), which was also used to study the valence state properties of the semiconductors. Fig. 7a displays the XPS survey spectrum of the CaFe_2_O_4_/g-C_3_N_4_/CNT composite. The major peaks of the Ca, Fe, O, C and N elements appeared in the XPS spectra. Fig. 7b displays the elemental scan of the Ca 2p element, where the major peaks at 346.4 eV and 350.2 eV correspond to the Ca 2p_3/2_ and Ca 2p_1/2_ spins, respectively.^24^ The elemental XPS spectrum of Fe 2p in Fig. 7c mainly shows peaks at 710.8 eV and 724.8 eV, representing the Fe 2p_3/2_ and Fe 2p_1/2_ spins, which indicate the oxidation of Fe was 3+ in CaFe_2_O_4_.^25^ The O 1s spectrum in Fig. 7d was deconvoluted into two main peaks at 529.7 eV and 531.8 eV, corresponding to the lattice oxygen in ferrites and surface-adsorbed OH groups, respectively.^26^ The C 1s peaks of the composite were located at 288.6 eV and 284.5 eV, which correspond to the C–N and C–C hybridization in the carbon matrix, respectively (Fig. 7e). The peaks observed at 398.7 eV and 398.5 eV are attributed to the sp^2^-hybridized nitrogen atoms in the triazine rings. Furthermore, the sharp peak observed at 401.4 eV corresponds to the strong binding of nitrogen with carbon atoms such as N–(C)_3_ (Fig. 7f).^27^ Thus, the structural analysis with XRD, FE-SEM, TEM, UV-vis DRS, and XPS suggests that the CaFe_2_O_4_/g-C_3_N_4_/CNT composite was successfully synthesized.

**Fig. 8:**
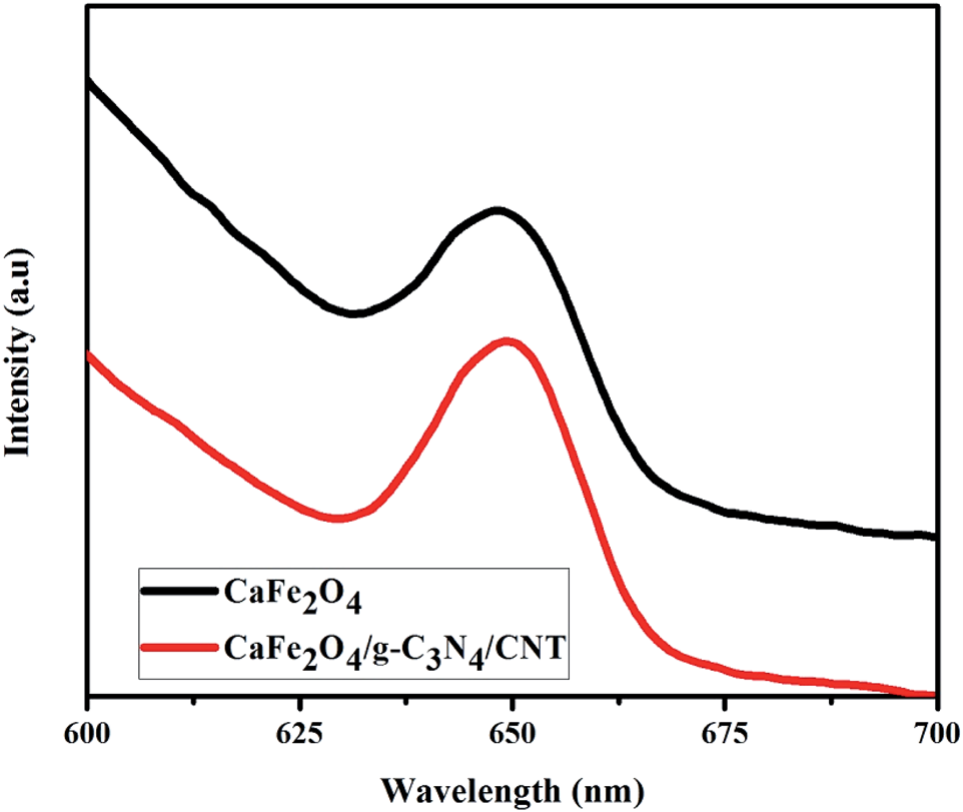
PL analysis of CaFe_2_O_4_ and CaFe_2_O_4_/g-C_3_N_4_/CNTs composite.

It is worth mentioning that highly proficient visible light-active photocatalytic reactions are mainly a result of fast separation efficiency, good redox reactions and high visible light absorption.^28^ The electron–hole separation and charge transfer properties of g-C_3_N_4_ and the CaFe_2_O_4_/g-C_3_N_4_/CNT composite was confirmed by PL analysis, and the data is shown in Fig. 8. g-C_3_N_4_ showed the highest PL peak intensity, which confirms that it has a very high recombination rate. However, the PL intensity of the CaFe_2_O_4_/g-C_3_N_4_/CNT composite was remarkably compared with that of g-C_3_N_4_. The lower recombination rate in the photocatalytic system can be beneficial for the complete degradation of pollutions within a minimum time.^29^

**Fig. 9:**
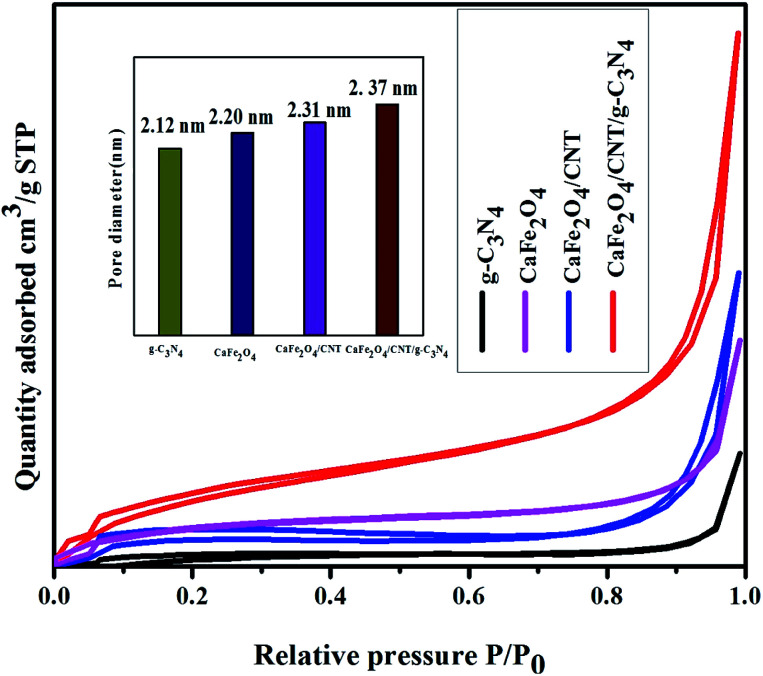
BET analysis of CaFe_2_O_4,_ g-C_3_N_4,_ CaFe_2_O_4_//CNTs, and CaFe_2_O_4_/g-C_3_N_4_/CNT composite.

The BET surface area and average pore size of CaFe_2_O_4_, g-C_3_N_4,_ CaFe_2_O_4_/CNTs, and the CaFe_2_O_4_/g-C_3_N_4_/CNT nanocomposite were obtained from N_2_ adsorption–desorption studies, as shown in Fig. 9. The N_2_ adsorption–desorption curves of the prepared samples exhibit type IV isotherms, indicating the pore size of the samples is mesoporous in nature using the BJH method. It was evident that the CaFe_2_O_4_/g-C_3_N_4_/CNTs photocatalyst exhibited the highest specific surface area, which is beneficial for higher photocatalytic activity.

**Fig. 10:**
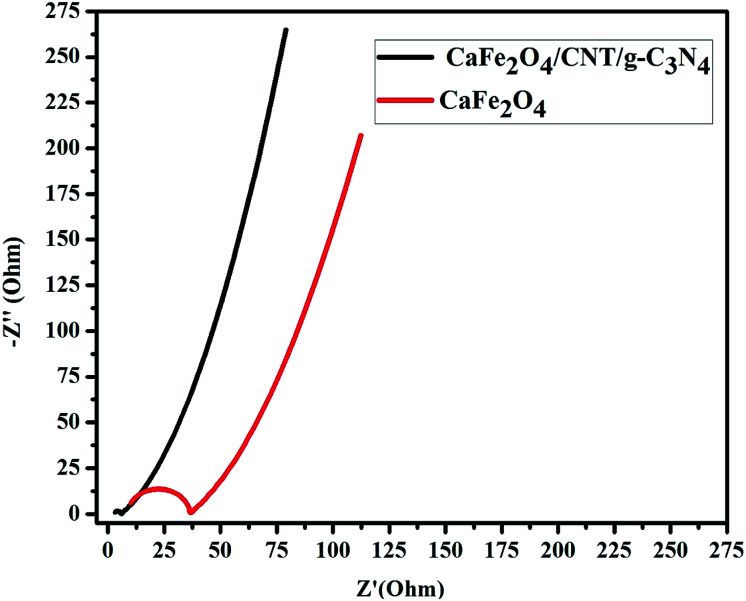
EIS spectra CaFe_2_O_4_ and CaFe_2_O_4_/g-C_3_N_4_/CNT composite.

The photogenerated charge carrier capability and separation nature of the composite were investigated by EIS measurements. Both CaFe_2_O_4_ and the CaFe_2_O_4_/g-C_3_N_4_/CNT composite exhibited semicircular Nyquist plots, but the diameter of the CaFe_2_O_4_/g-C_3_N_4_/CNT composite plot was smaller (Fig. 10), further confirming the faster interfacial charge transfer rate and outstanding separation rate of photogenerated charge carriers between g-C_3_N_4_ and CaFe_2_O_4_ due to the incorporation of CNT.

**Fig. 11:**
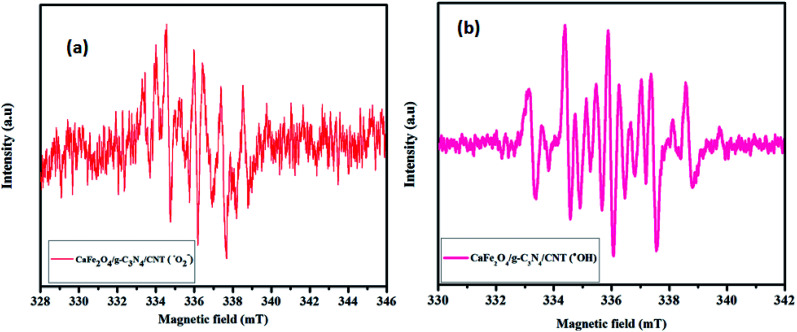
EPR spectrum of CaFe_2_O_4_/g-C_3_N_4_/CNT composite using DMPO.

The spin-trapping ESR measurements were used to confirm the presence of active oxidation species produced over the CaFe_2_O_4_/g-C_3_N_4_/CNT composite during the photocatalytic process, and the results are shown in Fig. 11. Under visible light irradiation, the characteristic peaks of DMPO–·O_2_^−^ and DMPO–·OH were clearly observed, and the peak intensities were obvious when the irradiation time was increased up to 3 min. The ESR results confirmed that the ·O_2_^−^ and ·OH radicals were mainly generated during the photocatalytic process.

### 3.1. Photocatalytic activities and stability tests

**Fig. 12:**
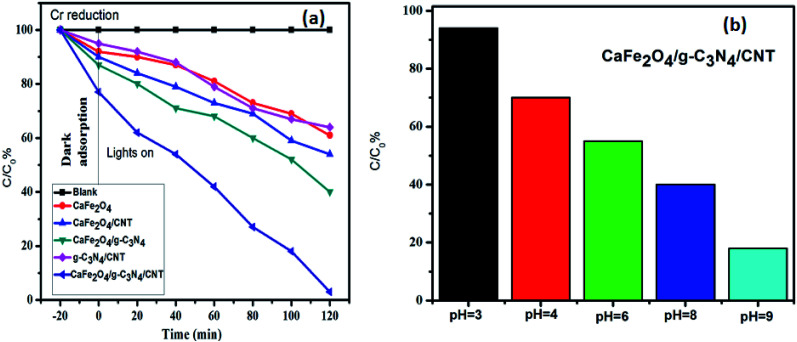
(a) Adsorption and visible light photocatalytic performance of the samples for the degradation of Cr(vi) and (b) effect of pH on the removal of Cr(vi) using the CaFe_2_O_4_/g-C_3_N_4_/CNTs composite.

**Fig. 13:**
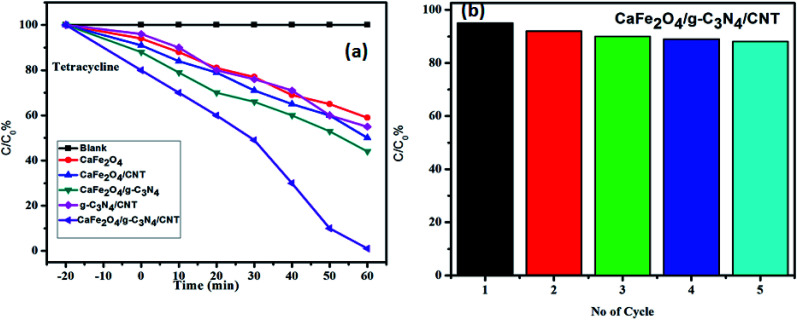
(a) Adsorption and visible light photocatalytic performance of the samples in the degradation of TC and (b) stability test of the CaFe_2_O_4_/g-C_3_N_4_/CNT composite in recycling degradation of TC.

**Fig. 14:**
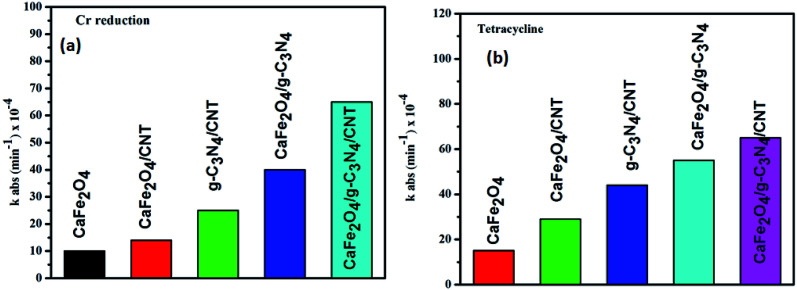
Photodegradation kinetics of Cr and TC over the CaFe_2_O_4_/g-C_3_N_4_/CNT composite under visible light.

The photocatalytic activity of g-C_3_N_4,_ CaFe_2_O_4_, and CaFe_2_O_4_/g-C_3_N_4_/CNT composite for the visible light degradation of Cr(vi) and TC is shown in Fig. 12a. The photolysis of Cr(vi) and TC under visible light without photocatalysts was negligible. Using the pure g-C_3_N_4_ and CaFe_2_O_4_, the degradation rate was not appreciable due to their high recombination rate of charge carriers. Furthermore, using the binary systems of g-C_3_N_4_/CNT and CaFe_2_O_4_/CNT, the degradation rate of Cr(vi) and TC moderately increased. We found that the introduction of CNT in the g-C_3_N_4_/CaFe_2_O_4_ heterojunction enhanced the degradation rate for Cr and TC, and the best degradation efficiency was achieved upon the incorporation of CNT in the system, which confirms the importance of CNT in the ternary structure. The CaFe_2_O_4_/g-C_3_N_4_/CNT composite exhibited a photocatalytic efficiency of 97% (at 120 min) for the degradation of Cr(vi) and 98% (at 60 min) for TC under visible light (Fig. 13a). As show in Fig. 13b, the photodegradation rate of the CaFe_2_O_4_/g-C_3_N_4_/CNT composite decreased slightly after five reaction cycles, indicating the excellent reusability of the photocatalytic material. The role of pH strongly interferes with the photocatalytic removal of Cr(vi); thus, the effect of pH was also studied in the pH range of 3–9 with the CaFe_2_O_4_/g-C_3_N_4_/CNT composite. At a higher pH, the photocatalytic reduction rate for Cr(vi) was significantly reduced (Fig. 12b) due to the formation of Cr_2_O_7_^2−^. At a lower pH value, Cr(vi) was easily removed due to the formation of HCrO^4−^ and the protonated surface of the catalyst, which is more favorable the Cr(vi) removal.^30^ Moreover, the reaction rate constant of the CaFe_2_O_4_/g-C_3_N_4_/CNTs ternary photocatalyst, as shown in Fig. 14, for the removal of Cr(vi) is 65 × 10^−4^ min^−1^ and removal of TC is 73 × 10^−4^ min^−1^.

### 3.2. Photocatalytic hydrogen production tests

**Fig. 15:**
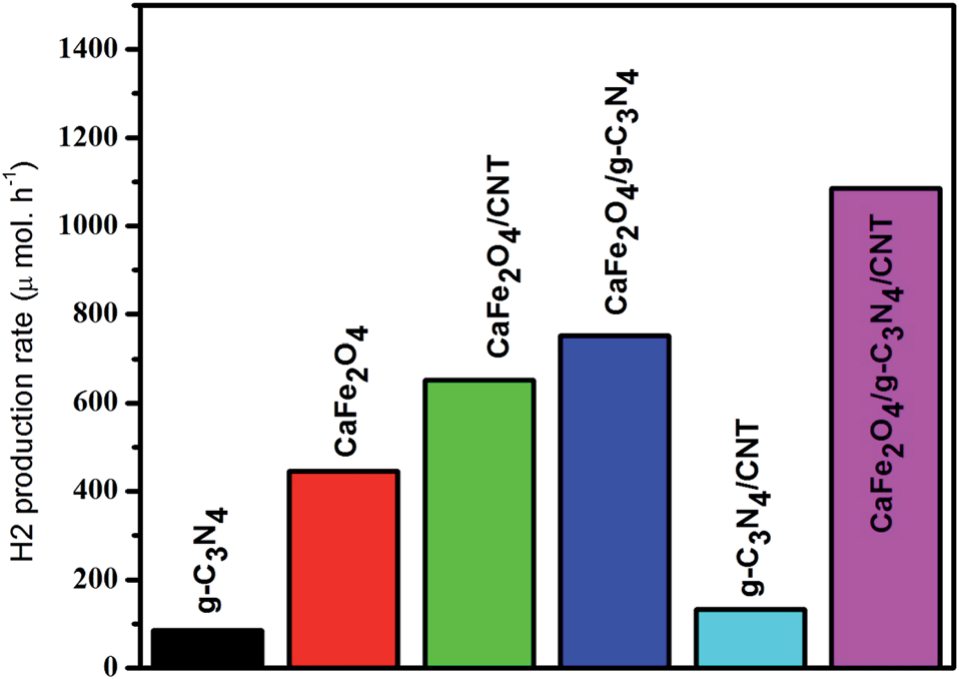
Time-dependent photocatalytic hydrogen production rate of the CaFe_2_O_4_/g-C_3_N_4_/CNT composite under visible light.

**Fig. 16:**
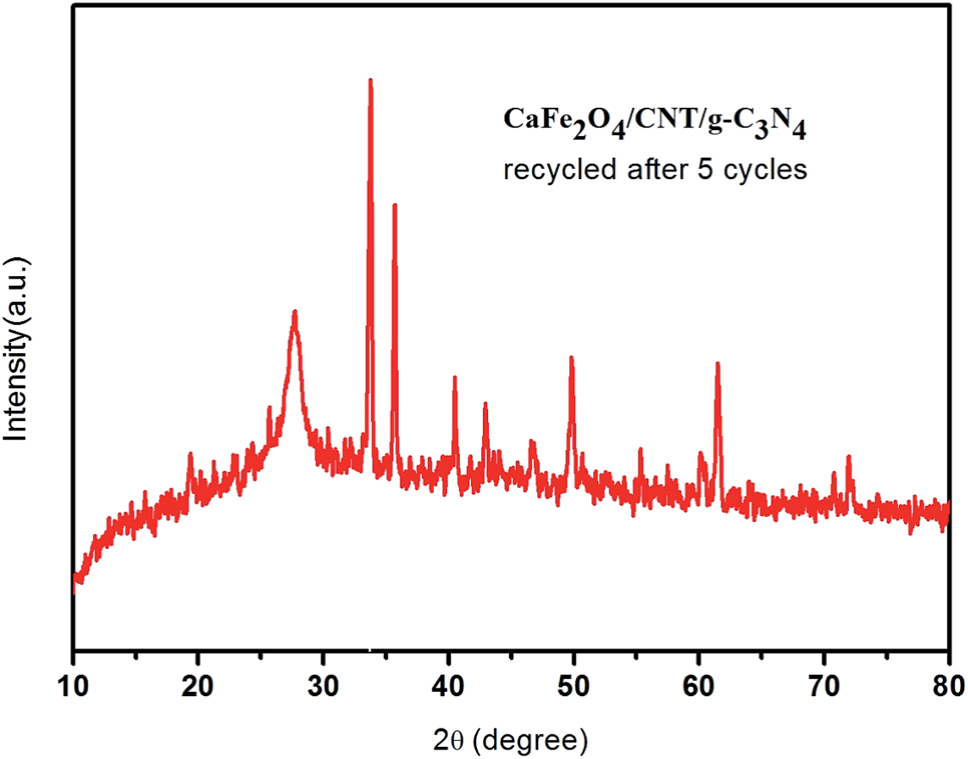
XRD spectrum of the reused CaFe_2_O_4_/g-C_3_N_4_/CNT composite towards hydrogen production after three cycles.

In this work, the feasibility of using the CaFe_2_O_4_/g-C_3_N_4_/CNT composite as a versatile photocatalyst for the production of hydrogen was studied. As shown in Fig. 15, the loading of CNT on g-C_3_N_4_ and CaFe_2_O_4_ resulted in a significant enhancement in the photocatalytic hydrogen production rate. Initially, with an increase in the loading of g-C_3_N_4_ in CaFe_2_O_4_, the hydrogen generation rate showed a remarkable improvement. In particular, the hydrogen generation rate of the CaFe_2_O_4_/g-C_3_N_4_/CNT composite sample increased to 1085 μmol h^−1^, which is about nearly 3 times higher than that of bare CaFe_2_O_4_ (417 μmol h^−1^). The AQY of CaFe_2_O_4_/g-C_3_N_4_/CNTs was calculated to be 7.2%. These results indicate that the CNT act as an effective co-catalyst for g-C_3_N_4_/CaFe_2_O_4_ and heterojunctions, and the loading ratio of CNT needs to be controlled within an appropriate range. Therefore, as a noble metal-free catalyst, CaFe_2_O_4_/g-C_3_N_4_/CNT composite can potentially be used as an economically feasible catalyst to replace precious metals such as Pt and Pd industrially to produce hydrogen. As shown in Fig. 16, to study the stability of the CaFe_2_O_4_/g-C_3_N_4_/CNT composite, cyclic runs for photocatalytic hydrogen production using the CaFe_2_O_4_/g-C_3_N_4_/CNT composite sample were performed. A slight decrease in the hydrogen production rate of the CaFe_2_O_4_/g-C_3_N_4_/CNT composite was observed in the last cycle, which confirms that the CaFe_2_O_4_/g-C_3_N_4_/CNT composite has excellent stability during the photocatalytic production of hydrogen besides the degradation reactions. Furthermore, the sample was collected after the cycle test and then characterized by XRD analysis. The XRD patterns of the reused catalyst exhibited no significant changes in the crystal structure after five consecutive cycles. Thus, the CaFe_2_O_4_/g-C_3_N_4_/CNT composite was determined to display good cyclic stability during the photocatalytic production of hydrogen.^31^ Using all the characterization results, the plausible photocatalytic degradation mechanism with the CaFe_2_O_4_/g-C_3_N_4_/CNT composite for a high degradation rate and hydrogen production was proposed. Also, a comparison of the present work with previous reported catalysts is presented in Table 1.

**Table 1:** Catalytic, photocatalytic and post-illumination activities of different catalysts

Photocatalyst	Target pollutant	Irradiation time (min)	Decomposition (%)	Ref.
Biochar-coupled g-C_3_N_4_	Cr(vi)	300	100	41
TiO_2_/g-C_3_N_4_@diatomite hybrid photocatalyst	Cr(vi)	300	100	42
N_2_-g-C_3_N_4_ photocatalyst	Bisphenol-A	120	79	43
g-C_3_N_4_/Na-bentonite composites	Cr(vi) and RhB	120	88.6	44
0D/2D bismuth molybdate homojunction	Cr(vi)	80	100	45
AgI/BiVO_4_ p–n junction photocatalyst	Tetracycline and Cr(vi)	100	70	46
CaFe_2_O_4_/g-C_3_N_4_/CNT composite	Tetracycline and Cr(vi)	120	98	This study

### 3.3. Photocatalytic mechanisms

Under visible light illumination, both CaFe_2_O_4_ and g-C_3_N_4_ can be quickly excited to generate the corresponding photoinduced electrons and holes due to their moderate band gaps of 1.55 eV and 1.55 eV, respectively. Since the *E*_CB_ value of g-C_3_N_4_ (−1.15 eV)^32^ is highly negative compared to that of CaFe_2_O_4_ (+1.57 eV), the ejected electrons towards the CB of g-C_3_N_4_ can be simply transferred to the CB of CaFe_2_O_4_. Meanwhile, the generated holes on the VB of CaFe_2_O_4_ may be transferred to the VB of g-C_3_N_4_ because the *E*_VB_ of CaFe_2_O_4_ (+1.98 eV) was more positive than that of g-C_3_N_4_ (+0.89 eV).^33^ Furthermore, the additional impact of the CNT results in the transfer of more electron–hole pairs through the effective linkage, leading to an improvement in the lifetime of the carriers. In this mode, the charge separation of photogenerated electron–hole pairs can be drastically enhanced and the recombination rate can be minimized, thus improving the photocatalytic ability of the CaFe_2_O_4_/g-C_3_N_4_/CNT composite.^34–40^ The possible photocatalytic mechanism for the photodegradation of Cr(vi) and TC as well as hydrogen generation using the ternary CaFe_2_O_4_/g-C_3_N_4_/CNT composite under visible light irradiation is proposed in Fig. S1.†

## 4. Conclusion

In conclusion, we demonstrated a simple hydrothermal strategy for the fabrication of a ternary CaFe_2_O_4_/g-C_3_N_4_/CNT composite. The addition of CNT in the system outstandingly promoted the separation of photogenerated charge carriers. The as-synthesized CaFe_2_O_4_/g-C_3_N_4_/CNT composite exhibited improved light-harvesting capability, more proficient charge transfer capability and improved hydrogen production performance. The hydrogen production rate of the optimal CaFe_2_O_4_/g-C_3_N_4_/CNT composite was superior to that of pure CaFe_2_O_4_ and previous works. Furthermore, the optimal CaFe_2_O_4_/g-C_3_N_4_/CNT composite photocatalyst exhibited excellent performances for the photodegradation of Cr(vi) and TC with high stability. This work may inspire the development of rare earth- and noble metal-free catalysts for the degradation of pollutants and production of hydrogen in the near future.

## Conflicts of interest

There are no conflicts of interest in this paper.

## Supplementary Material

RA-009-C9RA05005A-s001
