# Correction: Synthesis of a well-dispersed CaFe_2_O_4_/g-C_3_N_4_/CNT composite towards the degradation of toxic water pollutants under visible light

**DOI:** 10.1039/c9ra90097g

**Published:** 2019-12-20

**Authors:** Fei Liu, Shaocan Dong, Zhaoxiang Zhang, Xiaqing Li, Xiaodong Dai, Yanping Xin, Xuewu Wang, Kun Liu, Zhenhe Yuan, Zheng Zheng

**Affiliations:** Shengli College, China University of Petroleum Dongying Shandong 257061 China lf88888888lz@sina.com; Shengli Oilfield Company Postdoctoral Research Station, Sinopec Dongying Shandong 257000 China; Petroleum Engineering Technology Research Institute of Shengli Oilfield Company SINOPEC Dongying Shandong 257000 China

## Abstract

Correction for ‘Synthesis of a well-dispersed CaFe_2_O_4_/g-C_3_N_4_/CNT composite towards the degradation of toxic water pollutants under visible light’ by Fei Liu *et al.*, *RSC Adv.*, 2019, **9**, 25750–25761.

The authors regret that mistakes were made during the preparation of Fig. 1 in the published article. In the original article, Fig. 1 presented XRD data for CaFe_2_O_4_/CNT, which inadvertently duplicated the data for CaFe_2_O_4_. In addition, the spectra in the original figure do not match with the relevant discussion of Fig. 1 (on page 25753–25754) which referred to XRD patterns for g-C_3_N_4_, CaFe_2_O_4_ and CaFe_2_O_4_/g-C_3_N_4_/CNT composite. The correct image for Fig. 1 is shown below but no changes are required to the associated discussion of Fig. 1.

**Fig. 1:**
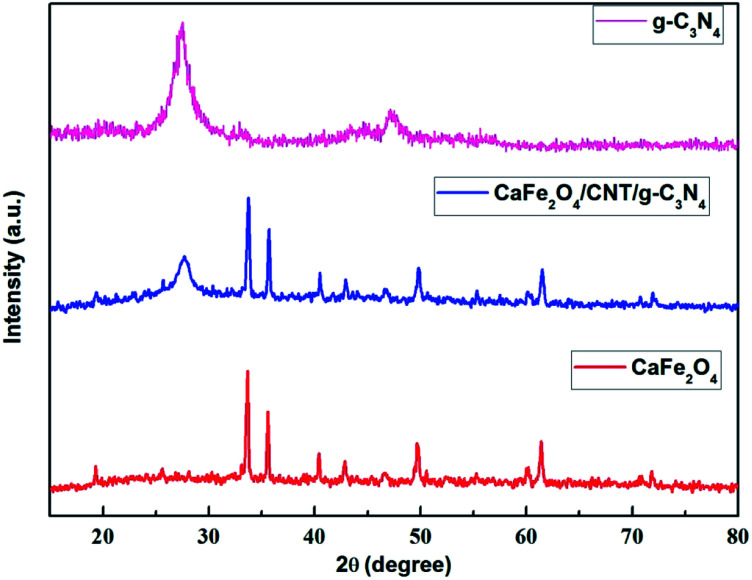
XRD patterns of the as-prepared CaFe_2_O_4_ and CaFe_2_O_4_/g-C_3_N_4_/CNT composite.

The Royal Society of Chemistry apologises for these errors and any consequent inconvenience to authors and readers.

## Supplementary Material

