# Supplementary figures and images for: Synthesis of a well-dispersed CaFe2O4/g-C3N4/CNT composite towards the degradation of toxic water pollutants under visible light
Source: RSC Adv. 2019 Aug 15;9(44):25750–61. doi: 10.1039/c9ra05005a (PMC9070011; doi:10.1039/c9ra05005a)

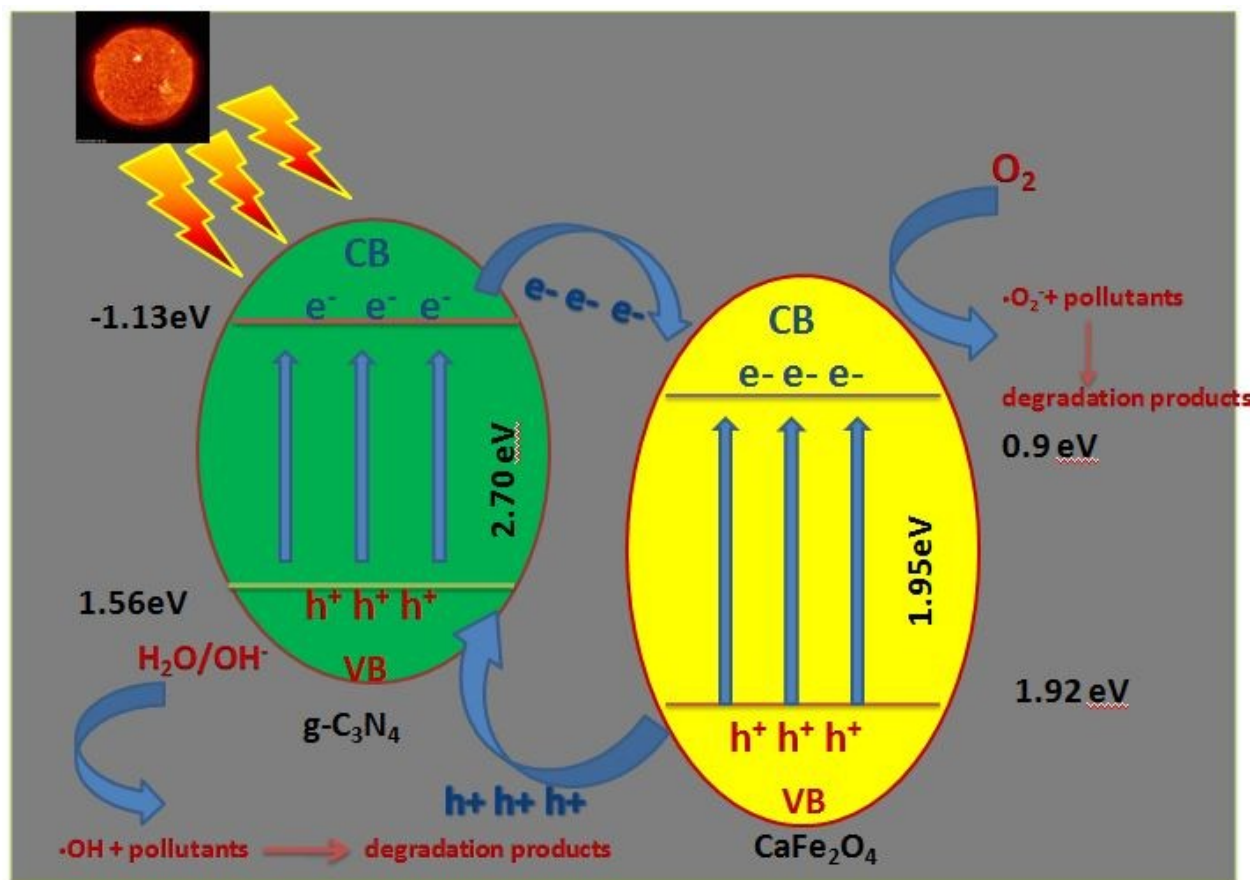

Fig.S1

Supplement: RA-009-C9RA05005A-s001 [file RA-009-C9RA05005A-s001.pdf]
